# Rapid measurement of intravoxel incoherent motion (IVIM) derived perfusion fraction for clinical magnetic resonance imaging

**DOI:** 10.1007/s10334-017-0656-6

**Published:** 2017-10-26

**Authors:** Emma M. Meeus, Jan Novak, Hamid Dehghani, Andrew C. Peet

**Affiliations:** 1 Physical Sciences of Imaging in Biomedical Sciences (PSIBS) Doctoral Training Centre, University of Birmingham, Birmingham, B15 2TT UK; 2 Institute of Cancer and Genomic Sciences, University of Birmingham, Birmingham, B15 2TT UK; 3 Department of Oncology, Birmingham Children’s Hospital, Steelhouse Lane, Birmingham, B4 6NH UK; 4 School of Computer Science, University of Birmingham, Birmingham, B15 2TT UK

**Keywords:** Diffusion weighted magnetic resonance imaging (DW-MRI), Biological models, Perfusion, Intravoxel incoherent motion (IVIM)

## Abstract

**Objective:**

This study aimed to investigate the reliability of intravoxel incoherent motion (IVIM) model derived parameters *D* and *f* and their dependence on *b* value distributions with a rapid three *b* value acquisition protocol.

**Materials and methods:**

Diffusion models for brain, kidney, and liver were assessed for bias, error, and reproducibility for the estimated IVIM parameters using *b* values 0 and 1000, and a *b* value between 200 and 900, at signal-to-noise ratios (SNR) 40, 55, and 80. Relative errors were used to estimate optimal *b* value distributions for each tissue scenario. Sixteen volunteers underwent brain DW-MRI, for which bias and coefficient of variation were determined in the grey matter.

**Results:**

Bias had a large influence in the estimation of *D* and *f* for the low-perfused brain model, particularly at lower *b* values, with the same trends being confirmed by in vivo imaging. Significant differences were demonstrated in vivo for estimation of *D* (*P* = 0.029) and *f* (*P* < 0.001) with [300,1000] and [500,1000] distributions. The effect of bias was considerably lower for the high-perfused models. The optimal *b* value distributions were estimated to be brain_500,1000_, kidney_300,1000_, and liver_200,1000_.

**Conclusion:**

IVIM parameters can be estimated using a rapid DW-MRI protocol, where the optimal *b* value distribution depends on tissue characteristics and compromise between bias and variability.

## Introduction

The acquisition of multi-*b* value diffusion-weighted magnetic resonance (DW-MRI) data and the bi-exponential signal decay observed in biological tissue have led to an increased number of studies using the intravoxel incoherent motion (IVIM) model [1, 2]. The IVIM model can be used to investigate the underlying tissue microenvironment and is based on the simultaneous assessment of two diffusion components. These correspond to the molecular diffusion in tissue (*D*) and diffusion affected by perfusion in the microcapillary network, often described as pseudo-diffusion (D*). The model also determines the fraction of signal arising from the microvascular network (*f*), known as perfusion fraction, which is thought to describe the vascularity of the tissue [1]. The IVIM model parameters have shown clinical value in the imaging of many different tumour types [3–5], as well as stroke [6, 7] and liver cirrhosis [8, 9]. The use of multi-*b* value DW-MRI has the potential to provide a single acquisition protocol for the non-invasive assessment of diffusion and perfusion in tissue.

The clinical adoption of the IVIM model has been hindered by practical issues and lack of consensus such as the number and choice of *b* values and requirements for a sufficient signal-to-noise ratio (SNR) level for accurate and reproducible post-processing [10–12]. Previous studies have demonstrated promising reproducibility for IVIM parameters *D* and *f* [3, 13], including a multi-centre brain study [14], whereas greater variability has been shown for the D* parameter. The application of the IVIM model has been well established in abdominal organs such as liver [11] and kidney [15], but applications in the brain [4, 16] have been more challenging due to the relatively low perfusion. However, recent studies have suggested that the use of the IVIM model, and the *D* and *f* parameters, has potential in brain tumour grading [4, 17] and stroke imaging [6, 18]. While these studies imply the clinical value of IVIM in the brain, the reported *f* values have been inconclusive with high variability, potentially owing to the different *b* value distributions used in the data acquisitions.

The translation of IVIM to clinical practice requires a DW-MRI protocol with a short acquisition time. One way to reduce the scanning time is to decrease the number of *b* values. Previously, a constrained (also known as segmented) IVIM fitting approach has been shown to provide the most robust IVIM parameters in many tissue types [10, 13, 19]. Using this methodology, the *D* and *f* parameters can be computed using high *b* values and *b* = 0 s/mm^2^. Additional low *b* values are required for the computation of the D* parameter. However, the challenges with the accuracy and reproducibility of D* in both brain and body, suggest that further evaluation is required to demonstrate its clinical value in terms of its reliability [12, 19–21]. Therefore, if only *D* and *f* are of clinical interest, the IVIM could be performed using a set of high *b* values, thereby minimising the time required for data acquisition.

The constrained fitting was recently used in a study by Conklin et al. [18], where the IVIM *f* parameter was estimated with a series of high *b* value combinations for brain tumour and stroke patients. The recommended *b* value distribution was chosen by comparison to the more commonly used fitting method (2-parameter fitting method [13]) in the brain. Although the similarity of the two fitting methods can indicate how many *b* values are required for comparable results, it is unable to assess the accuracy of the estimated IVIM parameters. Therefore, the purpose of this study was to use a minimum number of *b* values to minimise the scan duration and to assess the reliability of the estimated *D* and *f* parameters with different *b* value distributions, using simulated models with known ground truth values, and compare these results to IVIM data collected in vivo.

## Materials and methods

### Data simulations

**Fig. 1:**
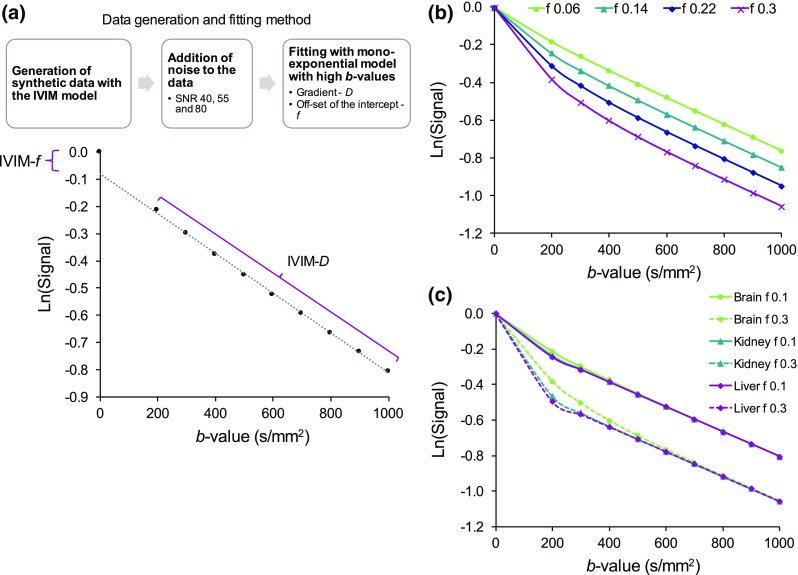
Description of **a** the mono-exponential fitting of the high *b* value diffusion signal to derive the IVIM *D* and *f* parameters from the fit gradient and off-set of the intercept to *S*(0) (signal at *b* = 0), respectively, and **b** the data signal decay at varying *f* values for the low-perfusion model (brain) and **c** comparison of the signals for different perfusion scenarios, respectively

All simulations and data analysis were implemented in MATLAB (MathWorks, Natick, MA, USA, v.2016b). The model data signal values were generated with Eq. 1 using a *b* value distribution: 0, 200, 300, 400, 500, 600, 700, 800, 900, 1000 s/mm^2^, as described in Fig. 1.

1 $${S ( b )} /S(0) = f \cdot \exp \left( { - bD^{*} } \right) + \left( {1 - f} \right) \cdot { \exp }\left( { - bD} \right)$$

Signal data sets were generated using a range of *f* values (0.06–0.30 in increments of 0.02) and three different D*/*D* ratios corresponding to previously reported ratios observed in the brain, kidney and liver (10, 20, and 70, respectively) [12]. The *D* parameter was fixed at 0.7 × 10^−3^ mm^2^/s and D* parameters used were brain: 7 × 10^−3^, kidney: 14 × 10^−3^ and liver 49 × 10^−3^ mm^2^/s. In this study, these models are also referred to as low-, medium-, and high-perfusion models, respectively.

Signal data for the different diffusion models and a series of *f* values is presented in Fig. 1b, c. Gaussian noise was introduced to the modelled data to simulate the Rician distribution of noise found in MR images using the in-built MATLAB function (Communications System toolbox). The Gaussian approximation is sufficient for the signal-to-noise ratio (SNR) levels of 40, 55, and 80, which were used to study the influence of noise on the estimated parameters [22]. SNR = 40 was chosen based on previous on-site measurements of diffusion-weighted MRI data [13] and the in vivo data presented here. The higher SNR levels 55 and 80 corresponded to approximately increasing the number of signal averages (NSA) from one to two and four, respectively. The same SNR level was used for all data points at different *b* values. The data simulations were performed using *N* = 1,000 random data iterations for each set of IVIM parameters.

### Volunteer population

A cohort of healthy young adult volunteers (*n* = 16, age 25–30, mean age 26 years) was scanned using a multi-*b* value diffusion-weighted imaging and *T*_1_-weighted imaging protocols. The protocols for this retrospective study were approved by the East Midlands – Derby Research Ethics Committee (REC 04/MRE04/41) operating under the rules of Declaration of Helsinki 1975 (and as revised in 1983), and informed consent was obtained from all volunteers.

### MR imaging

All MR imaging was performed on a Philips Achieva 3.0 Tesla (T) TX (Philips Healthcare, Best, the Netherlands) MRI scanner with a 32-multichannel receive head coil at Birmingham Children’s Hospital.

The diffusion-weighted MR protocol used a sensitivity-encoded (SENSE) approach with single-shot, spin-echo (EPI) sequence, with diffusion-weighted gradients applied in three orthogonal directions, of which an average diffusion-weighted image was derived. The protocol used TR/TE = 4000/91 ms, contiguous 3.5 mm thick axial slices, field-of-view (FOV) 240 × 240 mm and matrix size 96 × 96, which resulted in in-plane resolution of 2.5 × 2.5 mm. The *b* value distribution included values of 0, 300, 500, 1000 s/mm^2^ which were used in the IVIM analysis (full *b* value distribution: 0, 20, 40, 80, 110, 140, 170, 200, 300, 500, 1000 s/mm^2^). The scan duration was 2.12 min. The *T*_1_-weighted scan was performed with a spin-echo sequence with FOV 240 × 240 mm, matrix size 240 × 240, slice thickness 3.5 mm and TR/TE = 675/10 ms.

Additionally, four of the volunteer cases (*n* = 4) were scanned twice with the above DW-MRI protocol to assess the IVIM parameter repeatability.

### Data analysis

The data fitting was performed with the previously [13] reported constrained fitting method, shown in Fig. 1a. The fitting of the simulated diffusion-weighted signal was performed with *b* value distributions: [200,1000], [300,1000], [400,1000], [500,1000], [600,1000], [700,1000], [800,1000], and [900,1000]. Using the assumption that no IVIM effect is observed at high *b* values [23], the method allows the computation of *D* and *f* using the mono-exponential equation:

2 $${{S\left( b \right)} \mathord{\left/ {\vphantom {{S\left( b \right)} {S\left( 0 \right)}}} \right. \kern-0pt} {S\left( 0 \right)}} = { \exp }\left( { - bD} \right)$$

The *f* can be measured from the mono-exponential fit by extrapolating it to the *y*-intercept *S*(int) and taking the difference to the signal from *S*(0):

3 $${{f = 1 - S\left( {\text{int} } \right)} \mathord{\left/ {\vphantom {{f = 1 - S\left( {\text{int} } \right)} {S\left( 0 \right)}}} \right. \kern-0pt} {S\left( 0 \right)}}$$

The in vivo DW-MRI data (*n* = 16) was fitted using *b* value distributions [300,1000] and [500,1000], including the scans acquired for repeatability measurements. SNR levels of the data were determined using the standard NEMA method based on a difference image of two acquisitions, which is the recommended method for computing SNR when parallel imaging such as SENSE acceleration is used [24]. The SNR at *b* = 1000 s/mm^2^ was found to be in the range of 45 ± 8 and was similar across the brain.

The in vivo grey matter masks were created for each volunteer case with the brain extraction tool (BET) and FMRIB’s automated segmentation tool (FAST) in FMRIB Software Library package (Analysis Group, FMRIB, Oxford, UK, v. 5.0) using the *T*_1_-weighted images [25, 26]. The probabilistic tissue segmentation was performed for three classes, corresponding to grey matter, white matter, and cerebrospinal fluid (CSF). To assess the inclusion of only cortical grey matter and exclusion of sulcal CSF in the binary masks, partial volume tissue (PVE) segmentation was also performed for eight of the volunteer cases (*n* = 8). The PVE masks provided an estimation of the proportion of grey matter within the voxels (scale 0–1), and only voxels of value = 1 were included in the analysis, which corresponded to tissue fully representing grey matter with no partial volume of CSF or white matter. The *T*_1_-weighted images were acquired using the same spatial geometry as the DWI images, and both were visually inspected for any distortions. No further registration was performed at post-processing. The masks were adjusted for the size of the acquired DWI images using bi-linear interpolation, and a threshold of *T* = 0.7 was applied to remove any blurring effects around the edges. This further minimised the number of pixels affected by partial volume effects. For the analysis, the IVIM *D* and *f* values were extracted using the grey matter masks from three slices above the lateral ventricles.

Based on the extracted grey matter values, average histograms were computed for the IVIM parameters. The number of bins was based on the square root of the maximum number of data values extracted from the regions-of-interests (ROIs). The bin widths were computed for a range of zero to the maximum IVIM value. The same number of bins was used for all the cases and *b* value distributions, as well as for the IVIM values extracted with the PVE masks.

The same histogram methodology was applied to the simulated IVIM parameters.

The artwork in this manuscript was created with Microsoft Excel (Microsoft, Redmond, WA, USA, v.16.0) and Inkscape (GNU General Public Licence, v.0.91).

### Statistical analysis

All statistical analysis was performed in SPSS Statistics (IBM, Chicago, IL, USA, v.22). The following statistics were calculated for the data simulations and the estimated *D* and *f* parameters. Relative bias was determined from the difference between the true parameter (used in signal data generation) and the estimated parameter (computed from fitting of the signal data), which was normalised to the true parameter value:

4 $${\text{Relative bias}} = \frac{{\frac{1}{N}\mathop \sum \nolimits_{i = 1}^{N} \left( {x_{i} - X} \right)}}{X}$$

where *i* = number of iterations, *x*_i_ = estimated parameter and *X* = true parameter. Relative error (σ) was computed as the root mean square of the distance between the true parameter to the estimated parameter:

5 $${\text{Relative error}},\;\sigma = \frac{{\sqrt {\frac{1}{N}\mathop \sum \nolimits_{i = 1}^{N} \left( {x_{i} - X} \right)^{2} } }}{X}$$

Both relative bias and error were determined individually for each estimated parameter (*D*, *f*) rather than for the mean values over all data iterations. The overall relative error was computed from the individual parameter errors for each *b* value distribution using σ_*D*+*f*_ = σ_*D*_ + σ_*f*_. The overall error was used to make recommendations for the simulated tissue regions based on the smallest overall relative error.

The reproducibility of the estimated parameters was determined as a coefficient of variation from the ratio of the standard deviation to the mean of the estimated parameters:

6 $${\text{Coefficient of variation}} \left( \% \right) = \left( {\frac{{\sqrt {\frac{1}{N}\mathop \sum \nolimits_{i = 1}^{N} \left( {x_{i} - \bar{x}} \right)^{2} } }}{{\bar{x}}}} \right) \times 100$$

where $\bar{x}$ is the mean of the estimated parameter *D* or *f*.

For the in vivo data (*n* = 16), correlation analysis (Pearson correlation coefficient, *r*) was performed for the mean IVIM parameters in grey matter, to determine how the values were related between the *b* value distributions [300,1000] and [500,1000]. An analysis of variance (ANOVA) was performed to test if the estimated parameters differed significantly (*P* < 0.05). Bland–Altman analysis was used to determine the bias between the *b* value distributions. The repeatability of the IVIM parameters was tested using within-subject coefficient of variation (_w_CV%), which was the recommended statistic by the quantitative imaging biomarkers alliance [27] and has been applied in previous studies [3, 11, 19]. The _w_CV was computed with the root mean square method [28], using the paired DW-MRI data measurements (*n* = 4) and 4 × 4 ROIs (two from each measurement pair) of the same grey matter regions as used in the above analysis. To assess whether the IVIM values were influenced by CSF partial volume, ANOVA was performed for the IVIM histogram parameters derived with the probabilistic and PVE masks (*n* = 8) to determine any significant difference (*P* > 0.05).

## Results

### Model data

**Fig. 2:**
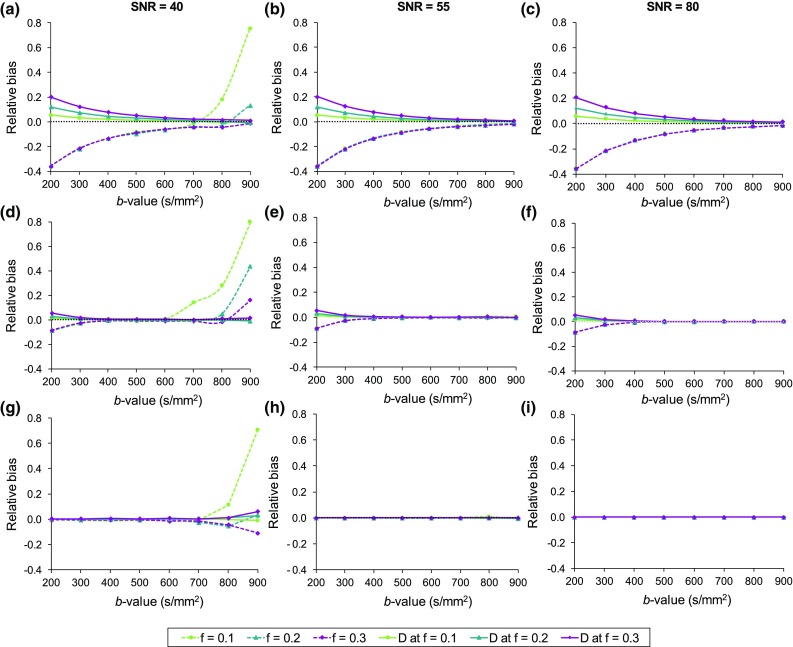
Relative bias results for **a**–**c** low-, **d**–**f** medium-, and **g**–**i** high-perfusion models at SNR levels 40, 55, and 80 as a function of *b* value. Results are presented for simulated *f* values of 0.1, 0.2, and 0.3 for both *D* and *f*. Bias = 0 is indicated by the black dashed line

The relative bias results for the estimated *D* and *f* parameters from the low-, medium-, and high-perfusion tissue models are presented in Fig. 2 for the different *b* value distributions and noise levels. Noise was found to influence the bias at SNR = 40 for high *b* values, whereas results at SNR = 55 and 80 resembled one another in magnitude and behaviour for all tissue models. The direction of bias was different for *D* and *f*, with positive and negative bias shown, respectively. At the higher SNR levels (55 and 80), the magnitude of the simulated *f* value was found to not affect the bias in estimation of *f*. However, at SNR = 40, noise influenced the simulated *f* values to a different extent at higher *b* values. The similarity of biases at SNR = 55 and 80, suggest that these present the intrinsic magnitude of biases from the fitting of the tissue models. Higher biases were observed for the lower perfusion models with lower D*/*D* ratio equating to lower degree of bi-exponential behaviour.

The choice of *b* value had a noticeable influence on the observed bias. The intrinsic bias of the models and estimated parameters was higher at low *b* value distributions, whereas noise affected the high *b* value distributions, although only for the *f* parameter. The bias of *f* for the low-perfusion model at SNR = 40 was −21.6 ± 0.27, −8.63 ± 0.8, and −5.77 ± 15.0% for [300,1000], [500,1000], and [800,1000] distributions, respectively. At SNR = 55 the biases were similar, but with reduced variability: −21.7 ± 0.07, 8.48 ± 0.05, and −2.62 ± 0.32%. Similarly for the *D* parameter at SNR = 40, the bias was 6.82 ± 3.31%, 2.72 ± 1.34%, and 0.61 ± 0.65% for [300,1000], [500,1000], and [800,1000] distributions, respectively. For the higher perfusion models, the bias was < 10% for *f*, apart from the high *b* value distributions (*b* = 700–900 s/mm^2^) at SNR = 40. The bias for the *D* parameter was < 6% for both higher perfusion models.

**Fig. 3:**
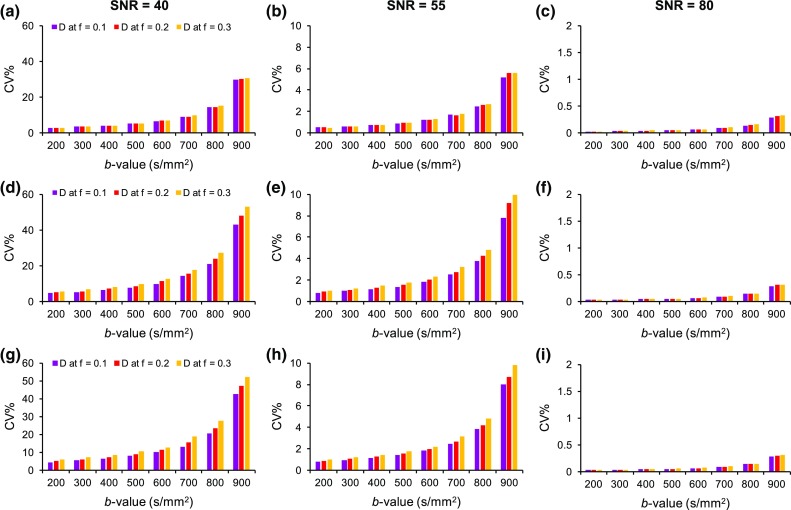
Reproducibility of diffusion coefficient, *D*, in low- (**a**–**c**), medium- (**d**–**f**), and high-perfusion (**g**–**i**) models at SNR levels 40, 55, and 80 for simulated *f* values: 0.1, 0.2, and 0.3

**Fig. 4:**
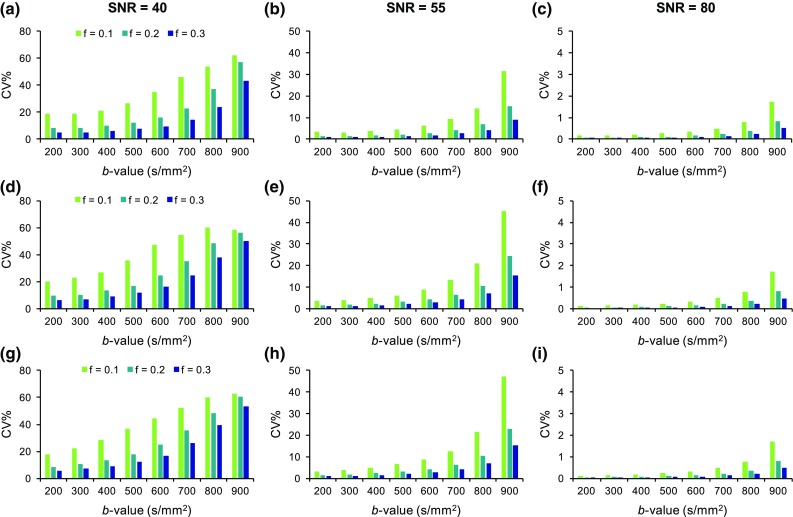
Reproducibility of perfusion fraction, *f*, in low- (**a**–**c**), medium- (**d**–**f**), and high-perfusion (**g**–**i**) models at SNR levels 40, 55, and 80 as a function of *b*-value for simulated *f* values: 0.1, 0.2, and 0.3

The reproducibility results for *D* and *f* parameters and the different tissue models are presented in Figs. 3 and 4, respectively. The variability of the estimated IVIM parameters was largely influenced by noise and dependent on the SNR level. The increase from SNR = 40 to SNR = 55 (NSA = 1 to NSA = 2) resulted in a noticeable improvement in the reproducibility of *D* and *f*, with a smaller improvement observed with the increase to SNR = 80. The coefficient of variation (%) of *f* for the low-perfusion model at SNR = 40 was 12.4 ± 7.4%, 17.6 ± 10.0, and 41.7 ± 12.6% for [300,1000], [500,1000], and [800,1000] distributions, respectively. At SNR = 55 these were reduced to: 2.23 ± 1.34, 3.14 ± 1.88, and 9.83 ± 6.04%. The different tissue models did not differ to a great extent in terms of their reproducibility for the *f* parameter, but the *D* parameter was found to be more reproducible with the low-perfusion model. Lower variability of *D* and *f* was observed with the use of lower *b* value distributions and the higher *f* values had better reproducibility compared to the low *f* values.

**Fig. 5:**
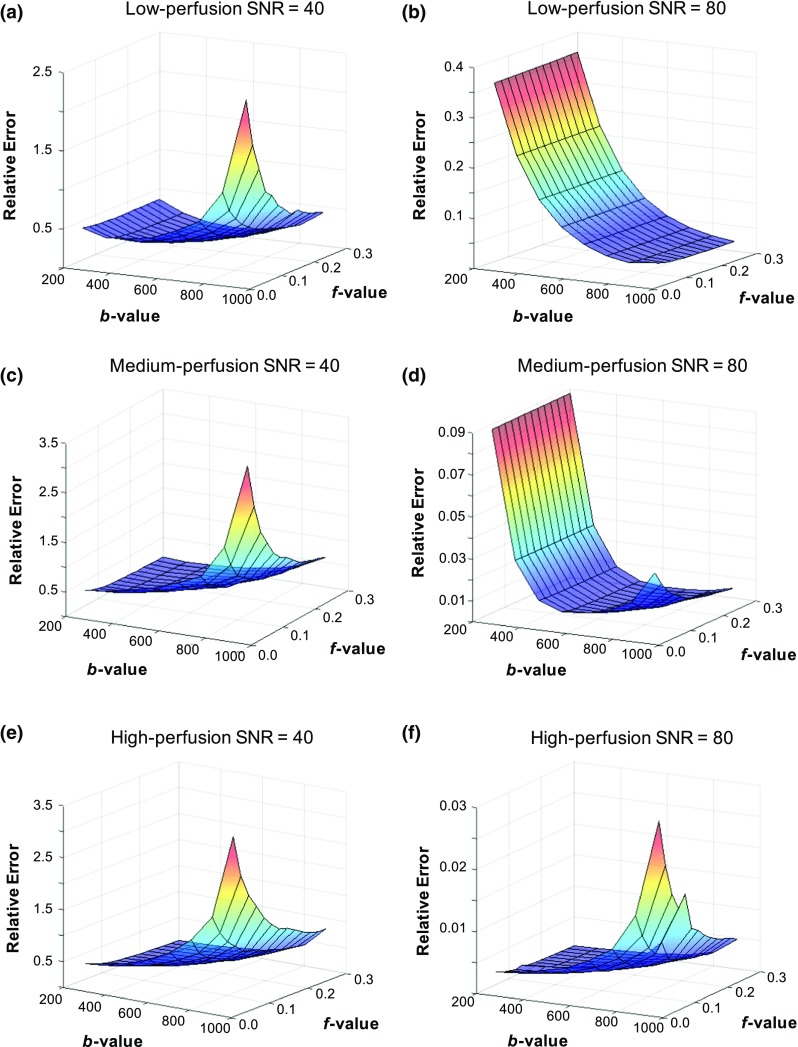
Contour plots of the relative error of perfusion fraction, *f*, with different *b* value distribution at SNR = 40 (**a**, **c**, **e**) and SNR = 80 (**b**, **d**, **f**) for the low- (**a**–**b**), medium- (**c**–**d**), and high-perfusion (**e**–**f**) models

The overall relative errors based on both *D* and *f* errors are summarised in Table 1 and presented visually in Fig. 5 for the *f* parameter. The overall error was largely influenced by the relative error of *f* with small contribution from the relative error of *D*. The relative error of *f* was greater than *D* in all cases. At SNR = 80 for low- and medium-perfusion models, the relative errors were higher at low *b* value distributions because of the bias, whereas negligible bias was observed with the high-perfusion model. At SNR = 40, noise had a larger influence on the estimated values compared to bias, resulting in higher relative errors at high *b* values. At SNR = 55, similar magnitude of contribution from bias and noise were seen for the low-perfusion model, whereas noise was the dominant contributor for the higher perfusion models.

**Table 1:** Overall relative error (± standard deviation) of the estimated *D* and *f* parameters

*b* values	Low-perfusion (brain)			Medium-perfusion (kidney)			High-perfusion (liver)		
	SNR = 40	SNR = 55	SNR = 80	SNR = 40	SNR = 55	SNR = 80	SNR = 40	SNR = 55	SNR = 80
[200,1000]	0.49 ± 0.04	0.47 ± 0.05	0.47 ± 0.05	0.22 ± 0.05	0.12 ± 0.01	0.12 ± 0.01	**0.17** ± **0.06**	**0.03** ± **0.01**	**0.001** ± **0.0005**
[300,1000]	0.32 ± 0.02	0.29 ± 0.03	0.29 ± 0.03	**0.21** ± **0.08**	**0.05** ± **0.01**	0.04 ± 0.004	0.21 ± 0.08	0.04 ± 0.01	0.001 ± 0.0006
[400,1000]	0.26 ± 0.05	0.18 ± 0.02	0.18 ± 0.02	0.26 ± 0.10	0.05 ± 0.02	0.01 ± 0.001	0.26 ± 0.09	0.05 ± 0.02	0.002 ± 0.0007
[500,1000]	**0.25** ± **0.08**	0.12 ± 0.01	0.11 ± 0.01	0.34 ± 0.13	0.06 ± 0.02	0.004 ± 0.0004	0.34 ± 0.13	0.06 ± 0.02	0.002 ± 0.001
[600,1000]	0.30 ± 0.12	**0.09** ± **0.01**	0.07 ± 0.01	0.45 ± 0.16	0.08 ± 0.03	**0.003** ± **0.002**	0.45 ± 0.17	0.08 ± 0.3	0.003 ± 0.001
[700,1000]	0.40 ± 0.15	0.09 ± 0.03	**0.05** ± **0.01**	0.61 ± 0.19	0.12 ± 0.05	0.004 ± 0.002	0.64 ± 0.23	0.12 ± 0.05	0.004 ± 0.002
[800,1000]	0.62 ± 0.23	0.13 ± 0.06	0.03 ± 0.003	0.92 ± 0.33	0.19 ± 0.08	0.007 ± 0.003	0.99 ± 0.33	0.19 ± 0.08	0.007 ± 0.003
[900,1000]	1.20 ± 0.52	0.26 ± 0.12	0.03 ± 0.003	1.78 ± 0.74	0.40 ± 0.15	0.01 ± 0.007	1.91 ± 0.67	0.41 ± 0.16	0.010 ± 0.007

Lowest relative errors are highlighted in bold for each SNR level and perfusion model

Based on the minimal overall and *f* relative errors, suggestions for optimal *b* value distributions were derived. The optimal *b* value distributions are listed in Table 2 for each perfusion model. At SNR = 40, the optimal *b* value distributions were [500,1000], [300,1000], and [200,1000] for the low-, medium-, and high-perfusion models, respectively. The relative errors of the estimated *f* parameters for these *b* value distributions were < 20% at SNR = 40, and < 10% for SNR = 55 and 80. The *b* value distribution for the low-perfusion model was higher because of the greater relative bias at the lower *b* values.

**Table 2:** Recommended *b* value distributions for computation of IVIM perfusion fraction, based on relative error of < 10%

Model	SNR	*b* value distribution	Relative error of *f* (%)	Overall relative error (%)
Low-perfusion	40	[500,1000]	18.7 ± 5.5^a^	24.8 ± 7.6^a^
	55	[600,1000], [700,1000]	< 10	< 10
	80	[500,1000]	< 10	< 10
Medium-perfusion	40	[300,1000]	15.3 ± 8.3^a^	21.3 ± 7.8^a^
	55	[300,1000] to [600,1000]	< 10	< 10
	80	≥ [300,1000]	< 10	< 10
High-perfusion	40	[200,1000]	12.1 ± 6.8^a^	17.2 ± 6.4^a^
	55	[200,1000] to [600,1000]	< 10	< 10
	80	[200,1000]	< 10	< 10

^a^Lowest relative error

### Volunteer data

The *b* value distributions [300,1000] and [500,1000] were investigated retrospectively for the volunteer cohort. The average values of *D* and *f* in the grey matter were 0.865 ± 0.05 (× 10^−3^ mm^2^/s) and 0.141 ± 0.02 with [500,1000], and 0.912 ± 0.05 (× 10^−3^ mm^2^/s) and 0.104 ± 0.01 with [300,1000], respectively. The higher *f* values and lower *D* values derived with the [500,1000] distribution agreed with the results from the low-perfusion model simulations.

**Fig. 6:**
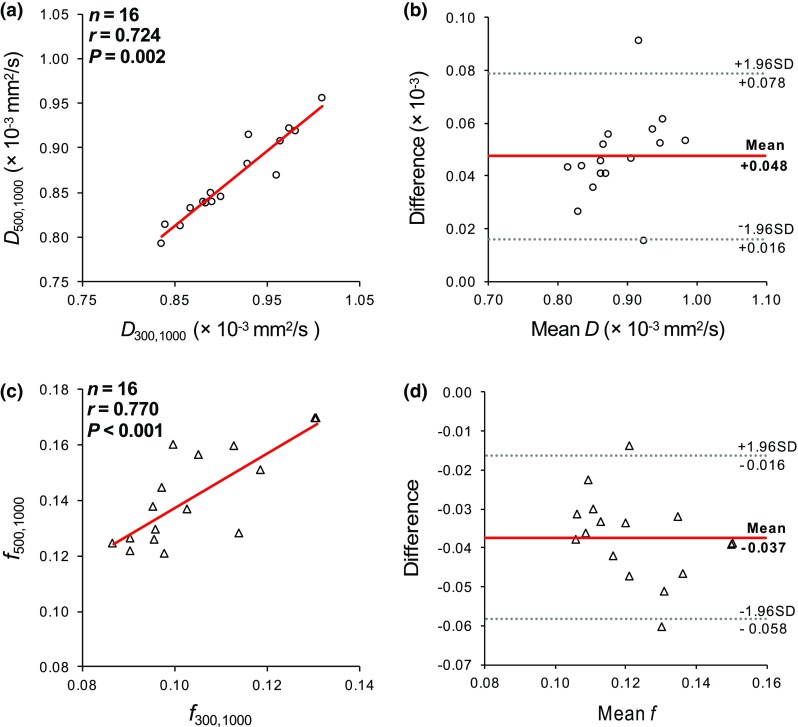
Correlation (**a**, **c**) and Bland–Altman (**b**, **d**) plots for *D* and *f* parameters in grey matter with *b* value distributions [500,1000] and [300,1000] for the volunteer cohort (*n* = 16). The red lines in the BA plots describe the mean difference of the values and the dashed lines the agreement range (95% confidence intervals)

The correlation and Bland–Altman analysis for the estimated *D* and *f* parameters are presented in Fig. 6. Significant correlations were established between both IVIM parameters derived with the different *b* value distributions, which indicated an existence of a linear relationship. Correlation of *r* = 0.724 (*P* = 0.002) was derived between the *D* parameters, and *r* = 0.770 (*P* < 0.001) between the *f* parameters. However, the estimation of *D* (*P* = 0.029) and *f* (*P* < 0.001) were significantly different between the [500,1000] and [300,1000] distributions. The agreement of methods, described by the Bland–Altman plots, showed a bias of 0.048 (× 10^−3^ mm^2^/s) and -0.037 for *D* and *f* parameters respectively. However, this only indicated the bias of estimating the IVIM parameters with [300,1000] in comparison to [500,1000]. The bias was smaller at the lower *f* values and greater towards the higher *f* values. In comparison to the simulated *f* = 0.1 value for the low-perfusion model at SNR = 40, the differences between the mean values for [300,1000] and [500,1000] were 0.015 (× 10^−3^ mm^2^/s) and −0.013 for *D* and *f* parameters, respectively. At *f* = 0.2, the differences were increased to 0.030 (× 10^−3^ mm^2^/s) and −0.025, although the relative bias remained the same.

**Fig. 7:**
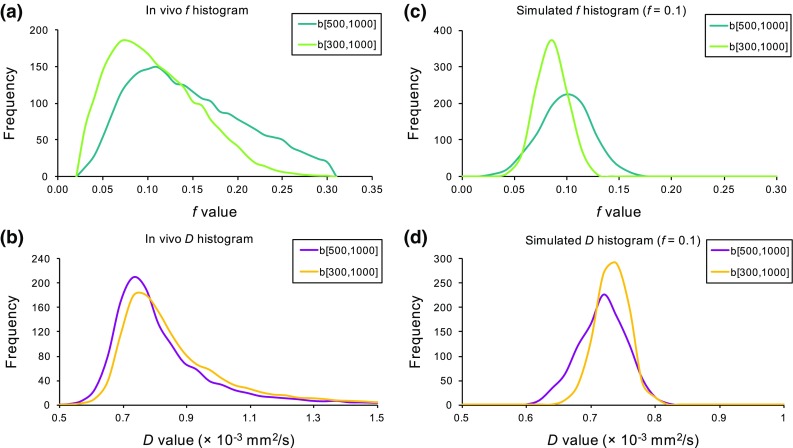
Histograms of IVIM diffusion coefficient and perfusion fraction for in vivo (**a**–**b**) and simulated (**c**–**d**) data with *b* value distributions [500,1000] and [300,1000]. The in vivo histograms are the average histograms derived for the grey matter regions of the volunteer cohort and the simulated histograms correspond to the estimated values from the low-perfusion model at SNR = 40 and *f* = 0.1

The average grey matter histograms for the IVIM parameters are presented in Fig. 7, together with histograms for the low-perfused brain model (where *f* = 0.1 at SNR = 40). Similar behaviour was observed between the in vivo and simulated data IVIM parameter histograms. The *f* histogram based on the [500,1000] distribution was shifted to higher *f* values compared to the [300,1000] distribution, with narrower distributions observed for the [300,1000] distribution.

The _w_CV was used to assess the repeatability of the IVIM parameters, which for the [500,1000] and [300,1000] distributions was 6.32 and 3.99% for *D*, and 15.3 and 10.8% for *f*, respectively. The values were similar to the ones depicted by the low-perfusion model at SNR = 40 (Figs. 3, 4), with small improvements seen with the use of [300,1000] over the [500,1000] distribution.

**Fig. 8:**
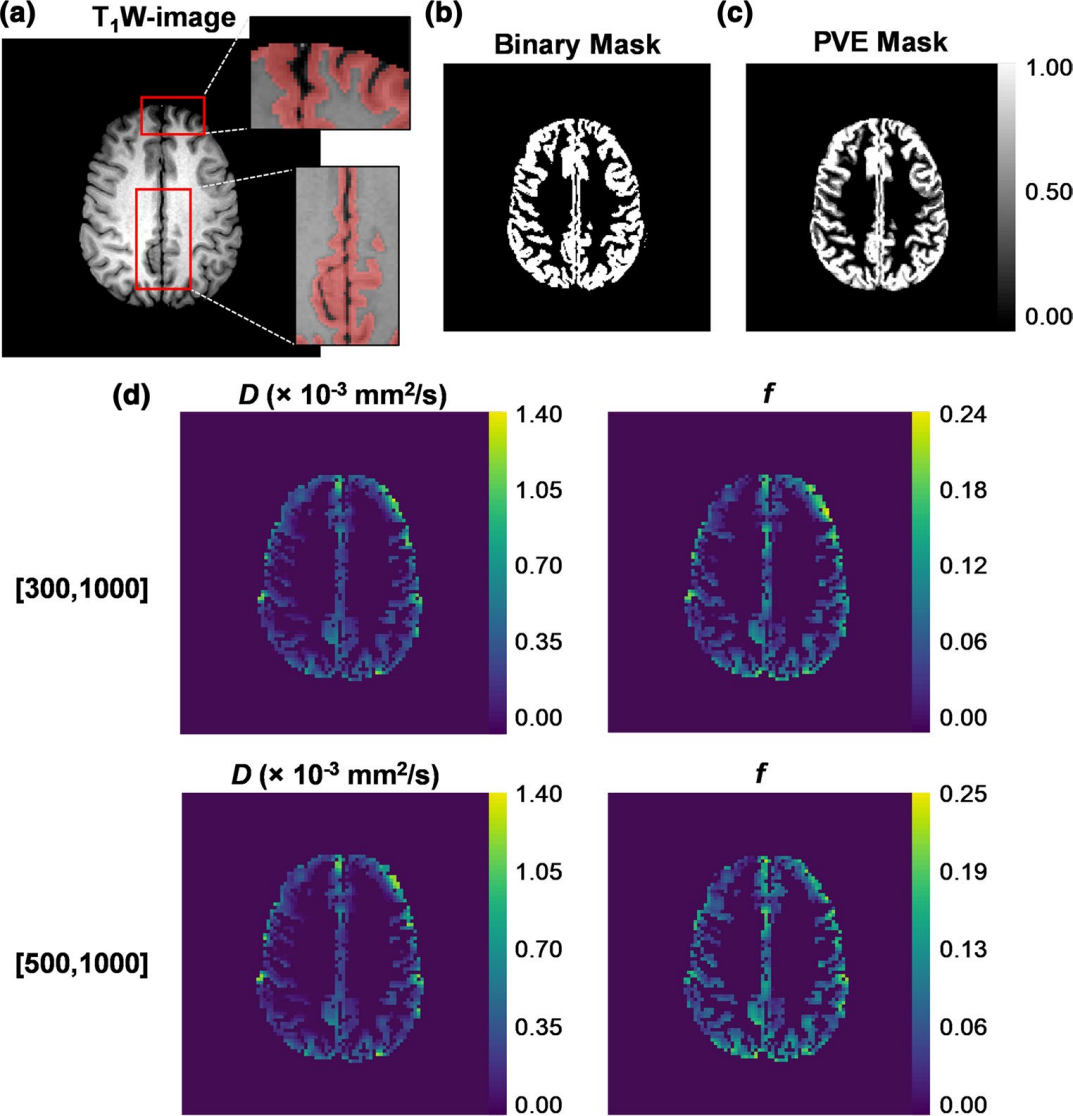
An example volunteer case with (**a**) *T*_1_-weighted image and overlaid binary grey matter mask regions showing the exclusion of CSF, (**b**) the binary mask, (**c**) the PVE mask, and (**d**) the extracted IVIM *D* (left) and *f* (right) parameter maps derived with the *b* value distributions [300,1000] and [500,1000]

The IVIM parameter histograms were compared to the ones derived with PVE masks for eight volunteer cases (*n* = 8). No significant differences were found between the mean, median, 10th and 90th percentiles of the IVIM parameters derived with the different masks for either [300,1000] or [500,1000] distributions. Example grey matter masks and IVIM parameter maps derived with *b* value distributions [300,1000] and [500,1000] are presented in Fig. 8 for a volunteer case. The overlaid regions on Fig. 8a showed that the sulcal CSF was successfully removed with the binary grey matter mask. The *f* maps derived with the [300,1000] and [500,1000] distributions were qualitatively similar, although differences in the magnitude of values could be observed, as depicted by the in vivo and simulation results.

## Discussion

The use of a simple fitting approach with a minimum number of *b* values was investigated to assess the feasibility of a rapid clinical application for determination of the IVIM perfusion fraction parameter. The accuracy and reliability of the IVIM parameters from different *b* value distributions were assessed using model simulations and confirmed using the in vivo image data. The model data simulations demonstrated that the optimal *b* value distributions for different tissue regions are dependent on the SNR level and the degree of perfusion influencing the diffusion signal.

The simulated tissue models were influenced by bias and noise to a different extent. Bias was found to be the dominant cause of higher relative errors at the low D*/*D* ratio and the low *b* value distributions. A similar effect was seen in a study by Conklin et al. [18], who demonstrated a negative bias in estimating *f* values in the brain by using a *b* value distribution [300,900], compared to distributions including more intermediate *b* values. The other cause for the higher relative errors was noise, which affected the estimation of perfusion fraction at higher *b* value distributions. This resulted in greater variability in extrapolating the linear fit back to the *y*-axis from the high *b* values. For the low-perfusion model, representing tissue perfusion of the brain, the effects of bias and noise were found to be minimised with the use of *b* value distribution [500,1000]. The intrinsic bias seen at the low perfusion meant that also at the higher SNR levels the recommended *b* value distribution was ≥ [500,1000].

The in vivo brain results indicated similar characteristics in the estimation of IVIM parameters as observed with the simulated model data. Higher *f* values were estimated with the [500,1000] distribution in comparison to the [300,1000] distribution, though the bias between the two *b* value distributions was higher in vivo compared to the simulated values where *f* = 0.1. However, this was expected with the variations observed in the D*/*D* ratio in vivo. The contributions from any potential partial volume effects due to the presence of CSF were minimised with the exclusion of sulcal CSF. This was confirmed by the comparison of IVIM values derived with the binary and PVE masks, which showed no difference in the distribution of the extracted IVIM values. The PVE masks were strictly generated and no voxels presenting tissue but grey matter were included. Nonetheless, the in vivo bias was within the observed range for the simulated *f* values (0.06–0.3) for the low-perfusion model, and confirms the presence of bias in low-perfused brain tissues. Therefore, consideration should be given to the impact of bias when choosing the *b* value distribution for the IVIM analysis, in particular for low-perfused tissues such as the brain or breast [3, 29, 30].

The medium- and high-perfusion models, representing tissues found in the abdomen, resulted in a substantial decrease in bias of estimating the perfusion fraction. Therefore, the more important factor for the optimal *b* value distribution was the variability arising from the noise. The estimation of *f* was found to be more sensitive to noise in comparison to *D*, although the increase in SNR level improved the reproducibility of both parameters considerably. The smaller contribution from bias meant that the lower *b* value distributions had lower relative errors in contrast to the low-perfusion model, with the optimal *b* value distributions for the medium-and high-perfusion models suggested to be [300,1000] and [200,1000], respectively.

The recommended *b* values from this study can be used to inform analysis of pre-existing data of different tissue types. The constrained fitting approach uses a *b* value threshold for the first fitting step on evaluating *D* and *f*, where perfusion effects are assumed to be negligible. Previously, thresholds of *b* = 100 s/mm^2^ for abdominal organs [31] and 200 s/mm^2^ for the brain [23] have been suggested, when using the constrained fitting. In our study, the use of *b* values < 500 s/mm^2^ for the low-perfusion model demonstrated high biases in estimation of *D* and *f* parameters, resulting in higher inaccuracies for any relatively low-perfused region. For higher perfused tissues, such as seen for the abdominal organs, the use of a lower *b* value threshold is reasonable due to the lower influence of bias. Although the *b* value recommendations were based on a relatively simple method of combining *D* and *f* errors, the aim was to provide *b* values that can guide the choice of *b* values, and minimise the intrinsic bias that arises from the fitting, even when using high quality data.

Previously reported IVIM parameters for different pathologies are listed in Table 3. For highly perfused tissues, such as reported for cirrhotic liver [8, 9, 32] hepatocellular carcinomas [5, 33], prostate cancer [34, 35] and many of the pancreas related pathologies [12, 36, 37], our results suggest that the use of a low *b* value can reduce the variability in estimating the perfusion fraction. In lower perfused tissues, such as reported for breast cancer [3, 29, 30], a higher *b* value can aid to reduce the bias.

**Table 3:** Previous IVIM studies of different pathologies and the reported IVIM parameters

Study	Pathology	No. of patients	D*/*D*	D*	*f*
Bisdas et al. [38]	Low-grade glioma	7	20.8	10.8	0.06
	High-grade glioma	15	54.7	41.6	0.11
Federau et al. [4]	Low-grade glioma	5	11.4	11.4	0.08
	High-grade glioma	16	5.85	11.7	0.13
Hu et al. [17]	Low-grade glioma	13	2.84	2.15	0.48
	High-grade glioma	29	5.35	2.71	0.29
Lin et al. [39]	Low-grade glioma	13	24.7	2.77	0.49
	High-grade glioma	11	29.0	5.10	0.40
Suo et al. [6]	Ischemic stroke	101	24.3	10.2	0.04
Cho et al. [3]	Breast cancer	14	10.7	15.0	0.13
Sigmund et al. [30]	Breast cancer	27	6.40	15.1	0.10
Hayashi et al. [32]	Cirrhotic liver	29	28.7	25.0	0.24
Luciani et al. [8]	Cirrhotic liver	12	51.2	61.0	0.30
Patel et al. [9]	Cirrhotic liver	14	26.8	27.9	0.25
Kuru et al. [34]	Prostate cancer	27	29.9	31.1	0.10
Ueda et al. [35]	Prostate cancer	63	11.5	7.48	0.23
Hectors et al. [5]	Hepatocellular carcinoma	25	47.1	64.1	0.18
Woo et al. [33]	Low-grade hepatocellular carcinoma	24	31.1	36.6	0.22
	High-grade hepatocellular carcinoma	18	32.5	32.3	0.19
Lemke et al. [12]	Pancreatic adenocarcinoma	23	17.4^a^	20.0^a^	0.09
Kang et al. [36]	Chronic pancreatitis	7	28.9	40.8	0.19
	Neuroendocrine tumour	17	39.4	43.7	0.30
	Pancreatic adenocarcinoma	39	19.6	22.3	0.12
	Intraductal papillary mucinous neoplasm	37	5.49	15.6	0.10
Klauss et al. [37]	Chronic pancreatitis	9	18.7^a^	20.0^a^	0.16
	Pancreatic adenocarcinoma	15	18.7^a^	20.0^a^	0.08

^a^ D* fixed at 20 × 10^−3^ mm^2^/s

The other low perfused region of clinical interest is the brain. Previous IVIM studies of brain gliomas have been inconclusive with the reported *f* values [4, 17, 38, 39]. A range of values were reported for low- (D* 2.15–11.4 × 10^−3^ mm^2^/s, *f* 0.06–0.49) and high-grade (D* 2.7–41.6 10^−3^ mm^2^/s, *f* 0.11–0.40) gliomas. Interestingly, the two studies [17, 39] including *b* values ≥ 1500 and up to 3500 s/mm^2^, reported relatively high *f* values for the brain (≥ 0.29), whereas the studies including *b* values ≤ 1300 s/mm^2^ [4, 38] reported much lower values (≤ 0.13). Tri-exponential fitting has been previously used for data with high *b* values (> 1000 s/mm^2^) in the brain [40], suggesting that using a bi-exponential fitting for higher *b* value data might result in under fitting and thus potential positive bias in estimation of the IVIM parameters. On the other hand, both the IVIM model and the tri-exponential model are unable to account for the non-Gaussian diffusion and noise observed at high *b* values [41]. An alternative method was introduced with the use of the IVIM kurtosis model, which can fully account for the non-Gaussian behaviour, as shown previously in a study by Iima et al. [29] investigating low-perfused breast tissue up to *b* values = 2500 s/mm^2^. Other challenges at the higher *b* values include the SNR level, which can be relatively low, and consequently increases the variability of the data, if not adjusted e.g. with the use of higher NSA. In the context of these issues, the use of the standard IVIM model at *b* values above 1000 s/mm^2^ might not be desirable. Overall, the differences in these studies make it challenging to assess the accuracy of the reported IVIM values, and therefore for studies in the relatively low perfused tissues, an estimate of the SNR level should be of importance as well as caution in the use of lower and higher *b* values, which can introduce bias to the results.

The increase of SNR by the increase in number of signal averages provided great improvements in the reliability of the estimated IVIM parameters. The increase from SNR = 40 to SNR = 55, corresponding to approximately an acquisition with one and two signal averages, increased the reproducibility for all the *b* value distributions. The improvement was less marked in going from an SNR = 55–80. Therefore, aiming for an SNR = 55 may be a reasonable compromise between reproducibility and length of acquisition, if the biological effects being investigated are large enough, such as seen between the low- and high-grade gliomas. Presence of small biological changes in tissue might require the use of higher SNR levels, where detection of the tissue properties can be improved with the better reproducibility of the *f* parameter.

Optimisation of *b* values for specific tissue regions with specific fitting methods have been reported previously [12, 42, 43]. The results from these studies include the D* parameter in the computation of the overall errors, which means that most of the contribution is likely to come from the D* due to its poor reliability [12, 13, 44]. This results in optimised D* parameter, but the variation of *f* might not have been taken into consideration. In our study, only the *D* and *f* parameters were considered, with larger contribution coming from the relative error of the *f* parameter. The recent interest in the *f* parameter for various brain pathologies, as well as for many types of cancer, indicates that a simple, but reliable approach is required for the transfer of IVIM to clinical imaging [45, 46].

The most used diffusion parameter in clinical practice remains the apparent diffusion coefficient (ADC). However, the use of *D* has shown better diagnostic performance in comparison to ADC in recent studies [33, 47]. Therefore, a clinical protocol with three *b* values could provide the option for computation of ADC, as well as the IVIM parameters *D* and *f*. The method used in this study can be easily adapted for clinical use by the introduction of a *b* value to an already routine protocol with *b* values 0 and 1000 with a small cost in scan duration. However, as suggested by the model simulations, awareness of the image quality and hence SNR is critical for the assessment of reliability of the derived IVIM parameters. Additional stability in the fitting of IVIM parameters can be achieved by increasing the number of averages, which were shown to provide large improvements on the results.

This study had some limitations. First, only three separate tissue models were investigated. While this provides a general guide on the use of optimal *b* values, variation in tissues creates a more complex scenario as indicated by the larger differences seen in vivo in comparison to the simulated results. Pathologies in the abdomen and the surrounding tissue have been found relative high perfused, implying that the recommended *b* value is likely to perform well for the whole imaged region. However, imaging in the abdomen can be affected by respiratory and cardiac motions, which must be assessed to ensure sufficient image quality for IVIM analysis. The *b* value choice for the brain is more complex, where bias is likely to play a greater role, and therefore, the use of higher *b* values should be considered. A second limitation is the importance of the noise level for the selection of *b* values. As with any imaging modality, data quality is important and an estimate of the SNR level can provide a good guidance on the reliability of the results and aid in choosing the optimal *b* values. Finally, a limitation of this study is the lack of availability of software for use in clinical practice, which is currently not offered on clinical workstations.

## Conclusion

This study demonstrated that IVIM parameters *D* and *f* can be estimated reliably with three *b* values. We have shown using model simulations that the optimal *b* value distribution depends on the diffusion and perfusion characteristics of the tissue and the compromise between bias and variability, which were validated using in vivo IVIM measurements. Recommendations for *b* values were made based on the model simulations, which can be used as a guide in future studies or for pre-existing data. With different clinical centres utilising different *b* value distributions, the results from this study can also aid in interpretation of differences seen between IVIM parameters of similar tissues.
